# Development of a Quartz Crystal Microbalance Biosensor with Aptamers as Bio-recognition Element

**DOI:** 10.3390/s100605859

**Published:** 2010-06-09

**Authors:** Chunyan Yao, Tangyou Zhu, Yongzhi Qi, Yuhui Zhao, Han Xia, Weiling Fu

**Affiliations:** 1 Department of Laboratory Medicine, Southwest Hospital, Third Military Medical University, Chongqing 400038, China; E-Mails yao_yao24@yahoo.com (C.-Y.Y.); zyhflyff@yahoo.com.cn (Y.-H.Z.); hxia16@gmail.com (H.X.); 2 Department of Dermatology, Daping Hospital, Third Military Medical University, Chongqing 400042, China; E-Mail: tangyouzhu@tom.com; 3 Department of Laboratory Medicine, the General Navy Hospital, Beijing 100037, China; E-Mail: qyz_2002_zyq@sohu.com

**Keywords:** quartz crystal microbalance, biosensor, aptamer, antibody, IgE

## Abstract

The ultimate goal in any biosensor development project is its use for actual sample detection. Recently, there has been an interest in biosensors with aptamers as bio-recognition elements, but reported examples all deal with standards, not human serum. In order to verify the differences of aptamer-based biosensor and antibody-based biosensor in clinical detection, a comparison of the performance of aptamer-based and antibody-based quartz crystal microbalance (QCM) biosensors for the detection of immunoglobulin E (IgE) in human serum was carried out. Aptamers (or antibodies) specific to IgE were immobilized on the gold surface of a quartz crystal. The frequency shifts of the QCM were measured. The linear range with the antibody (10–240 μg/L) compared to that of the aptamer (2.5–200 μg/L), but a lower detection limit could be observed in the aptamer-based biosensor. The reproducibility of the two biosensors was comparable. The aptamers were equivalent or superior to antibodies in terms of specificity and sensitivity. In addition, the aptamer receptors could tolerate repeated affine layer regeneration after ligand binding and recycling of the biosensor with little loss of sensitivity. When stored for three weeks, the frequency shifts of the aptamer-coated crystals were all greater than 90% of those on the response at the first day.

## 1. Introduction

Systematic evolution of ligands by exponential enrichment (SELEX) is an iterative selection procedure used to identify oligonucleotides with desired properties. The SELEX-derived aptamers generally have a high degree of similarity to antigen–antibody binding in both their specificity and affinity. Aptamers are synthetic, single-stranded DNA or RNA molecules that fold up into unique 3-D structures, which allows them to specifically bind to other target molecules. Since their discovery about 20 years ago [1,2], aptamers have exhibited high affinity to their targets with Kd values in the low nanomolar to picomolar range. Closely related isoforms, and even different conformational states of the same target molecule can be discriminated by using the aptamers with high specificity [3,4]. Due to these characteristics, aptamers have been successfully used in different areas of biotechnology such as purification processes [5], target validation [6], drug discovery [7], diagnostics [8], and therapy [9,10].

Recently, the stability and convenience of DNA aptamers have been exploited in diagnostic applications including biosensors [11,12] and flow-cytometry assays [13]. In comparison to antibodies, aptamer receptors have a number of advantages. The main advantage is the avoidance of the use of animals for their production. Moreover, the aptamer selection process can be manipulated to obtain aptamers that bind to a specific region of the target and with specific binding properties under different binding conditions. After selection, aptamers are produced by chemical synthesis and can be purified to a very high degree thus eliminating the batch-to-batch variation found when using antibodies. At the same time, modifications in the aptamer can be introduced to enhance the stability, affinity and specificity of the molecules. Finally, while many antibodies are temperature-sensitive and can denature upon contact with surfaces, leading to limited shelf lives and possible compromise of assay integrity, aptamers are stable to long-term storage [14–17]. Aptamers usually retain their binding and inhibitory behavior, even after immobilization on carrier material [18]. Aptamers can be transported at ambient temperature, and undergo reversible denaturation. Consequently, aptamers offer a useful alternative to antibodies as sensing molecules, especially in the development of biosensors for chemicals [19–21].

The key issue in the development of aptamer-based sensors is to convert target recognition into a measurable signal. QCM crystal transducers are known to be highly precise, stable oscillators and are capable of detecting subnanogram mass changes [22]. Several authors have reported direct comparisons between aptamers and antibodies specific for the same target when used as bio-recognition element in biosensors. An anti-IgE DNA aptamer was compared with the monoclonal antibody for the same target (IgE) in a quartz crystal biosensor [20]. The two receptors both achieved a detection limit of 0.5 nM for IgE, but the aptamer-based one showed a 10-fold extended linear range. The same kind of comparison has been performed on a piezoelectric biosensor using an RNA aptamer specific for HIV-1 Tat protein and the corresponding monoclonal antibody [21]. In this case an extended linear range was obtained with the antibody-based biosensor, but the aptamer-based sensor showed better sensitivity. The reproducibility of the two biosensors was comparable and a good specificity was observed with both the receptors.

We have set up a QCM immunosensor platform and successfully quantified human chorionic gonadotropin [23] and urinary protein [24]. The ultimate goal in biosensor development is to be able to use for clinical detection. Recently, there has been an interest in biosensors with aptamers as bio-recognition elements, but the reports all deal with standards. To the best of our knowledge, there are no reports of biosensors for detection of human serum. In a previous study, we constructed an aptamer biosensor and used it for serum detection [25]. In order to verify the differences between aptamer-based antibody-based biosensors in clinical detection, a comparison between the two methods was done and the main analytical parameters of the two biosensors, such as sensitivity, selectivity and reproducibility, have been studied.

## 2. Results and Discussion

### 2.1. Aptamer-Based Biosensor *vs.* Antibody-Based Biosensor: Comparison of Sensitivity

We compared the aptamer-based biosensor with the antibody-based biosensor with respect to achieved sensitivity and selectivity. The aptamer and antibody-coated crystals were incubated in IgE solutions in a range from 2.5–250 μg/L, both aptamer and antibody-based crystals showed typical binding capacity saturation. Theaptamer-based biosensor displayed signal saturation at the concentration of 200 μg/L IgE. The antibody-based biosensor performed similarly, but not exhibiting saturation below a concentration of 240 μg/L IgE. Although aptamers were likely to be immobilized in a denser arrangement than antibodies due to their smaller size, signal saturation did not shift to higher concentrations. This effect may be caused by steric hindrance between bound analyte molecules. The antibody-based biosensor generated significantly lower detection signals (ΔF), possibly caused by partial denaturation of the immobilized antibodies on the surface of crystals, leading to a decreasing number of correctly folded antibodies being available for specific analyte recognition.

Concerning the limit of detection, aptamers were proved to be superior compared to antibodies. The limit of detection (S/N, >3) was measured on 20 consecutive negative controls. The antibody-based biosensor was able to specifically detect IgE at a minimum concentration of 10 μg/L. In addition, specific analyte recognition by the aptamer-based biosensor could be observed down to a concentration of 2.5 μg/L in the binding assay. This result most likely reflected the dense and highly ordered nature of the aptamer receptor layer. The reaction time to reach equilibrium for both biosensors was 15 min. In a previous approach, anti-IgE antibodies and aptamers were compared as receptor molecules using a quartz crystal microbalance biosensor. Both receptor types detected IgE specifically at a minimum concentration of 95 μg/L [20]. The different sensitivity in that work could be partly attributed to the bigger gold surface (a diameter of 8 mm) of the PZ crystal they used. This usually results in a lower sensitivity. The aptamers they used were modified and had a longer sequence, that maybe another reason for the different sensitivity. This sensitivity is comparable or better than that of other reported aptamer-based analytical methods for IgE detection (Table 1).

### 2.2. Comparison of Imprecision

Imprecision data for the determination of IgE (2.5–200 μg/L) by the aptamer or antibody-based biosensor was compared intraassay and interassay. For every concentration, tests were repeated 20 times in one day for intraassay and repeated on 20 consecutive days in the same manner (mean of three duplicates per day) for interassay reproducibility. The mean intraassay and interassay CV of aptamer-based biosensor were 4.14% and 5.95%, respectively. Similarly, the intraassay and interassay CV of the antibody-based biosensor were 4.18% and 6.13%. Variable surface coverage between manually produced sensing elements might account for this precision difference. Large-scale, automated fabrication of aptamer biosensors would likely yield much more uniform surface coverage and a correspondingly lower CV.

### 2.3. Accuracy of the QCM Biosensor

**Figure 1.**
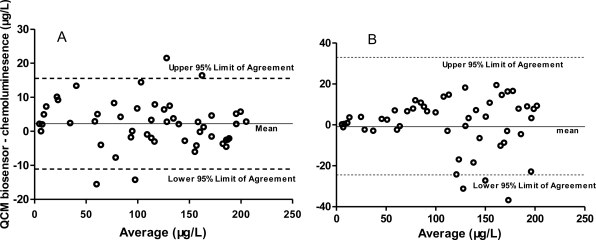
Bland-Altman difference plot for IgE detection results by QCM biosensor and chemoluminesence method. On the horizontal axis, plot the mean of results by the two studied methods. (A) Bland-Altman difference plot comparing IgE detection results obtained with aptamer-based QCM biosensor against Chemoluminesence (Beckman). (B) Bland-Altman difference plot comparing IgE detection results obtained with antibody-based QCM biosensor against Chemoluminesence. The solid line represents the mean difference in quantitative measurement of IgE between the two methods, and the dashed lines are mean ±1.96 SD.

We further tried IgE detection in human serum containing a variety of proteins, including different types of immunoglobulins. IgE concentrations in clinical human serum samples were simultaneously measured by the QCM biosensor and the chemoluminescence method. Mean values by the aptamer-based QCM biosensor, antibody-based QCM biosensor and chemoluminescence in 50 clinical human serum samples were 64.0, 62.6 and 64.9 μg/L, respectively, with ranges of 3–215, 5–234 and 5–208 μg/L. To investigate the correlation of the QCM biosensor with the chemoluminescence method, the Bland-Altman difference plot analysis for the clinical sample detection results was done (Figure 1). A Bland-Altman difference plot analysis for the aptamer-based QCM biosensor showed a mean difference (QCM minus chemoluminescence) of 2.12 μg/L, and the limits of agreement (d − 1.96S to d + 1.96S, −11.12 to 15.56 μg/L) were sufficiently narrow, suggesting good consistency and clinical comparability between these two methods. However, the antibody-based QCM biosensor had a wider range of agreement limit. The result showed that the IgE concentration in clinical serum samples was positively related to the frequency shift of the QCM biosensor. The consistency between QCM aptamer biosensor and chemiluminescence method is good, indicating that it is possible to detect IgE in clinical serum samples using the QCM aptamer biosensor and the analytical sensitivity and accuracy of the QCM biosensor is sufficient for the detection of IgE from serum samples.

### 2.4. Comparison of Selectivity

**Figure 2.**
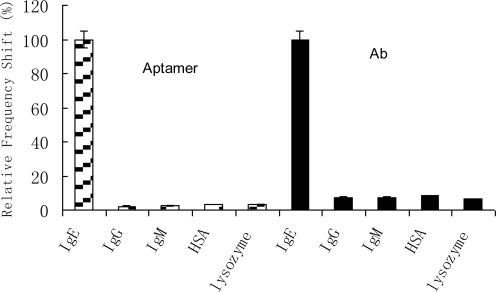
Comparison of the detection specificity between aptamer-based biosensor and antibody-based biosensor. The HSA, IgG and IgM were tested at a concentration of 500 mg/L and diluted in the same buffer (10 μM Tris-HCl buffer, pH 7.9) (interaction time: 15 min). The lysozyme was tested at a concentration of 1 mg/L. The testing concentration of IgE was 100 μg/L.

One of the greatest challenges a protein biosensor faces is nonspecific binding, which results in high background signals and therefore decreasing assay sensitivity. In this respect, a comparison between the signal intensities of specific IgE recognition with nonspecific protein binding again emphasized the superiority of aptamers. To test the specificity of the aptamer, proteins with different molecular weight and isoelectric point were used. The interfering proteins that were tested included HSA, lysozyme, IgG, and IgM. These proteins were chosen to cover the range of possible interfering molecules in the detection of IgE. HSA has a much smaller molecular weight (MW 66,000) with respect to IgE (MW 190,000), whereas IgG is structurally similar to IgE and thus can serve as a good test protein for the aptamer binding specificity. The concentration of the interfering proteins was chosen to be 500 mg/L; it was a very high concentration compared with the tested IgE. The relative responses of the interferences, as seen in Figure 2, were insignificant in comparison to the specific binding response with IgE. The testing concentration of IgE was chosen as 100 μg/L since at this concentration the specific signal for IgE resulted in a lower CV. These experiments demonstrated the high specificity of the sensor with the immobilized aptamers, as a significant frequency shifts were obtained only when testing the specific protein. The cross-reactivity of the aptamers was thus seen to be less than 5% for all the interferences considered. The relatively low cross-reactivity of aptamer toward HSA, IgG and IgM considered here was very encouraging, considering the high percentage of these proteins in serum. Lysozyme is a particularly good control in this experiment, because it has a very high basic charge (pI = 9.1) and is known to bind nonspecifically to nucleic acids. The results demonstrated that the contribution to the binding of the mere electrostatic interaction was reduced when considering the selected aptamer with its secondary structure. Therefore, nonspecific binding did not affect the analytical data. In contrast, binding assays carried out on antibody-coated crystals resulted in higher signal background and therefore lower selectivity. It could be clearly demonstrated that aptamers showed higher selectivity compared to antibodies, indicating strikingly higher specificities.

### 2.5. Comparison of Regeneration and Long-Time Stability

The regeneration of binding surfaces is important for reusable biosensors but it often difficult to achieve. We tried to regenerate the biosensor surface after binding of IgE by rinsing the antibody-coated crystals with 0.2 mmol/L glycine/HCl (pH 2.8), or 1.2 mmol/L NaOH, the common regents for dissociating antibody-antigen complexes. This resulted in a complete release of the analyte. However, all subsequent injection of IgE only led to reduced frequency changes. Apparently, the receptor layer was either irreversibly damaged or the antibodies were unfolded. The refolding of the antibodies was too inefficient to be effective before subsequent injection of the analyte. Although the antibody-based biosensor could be reusable, a loss of activity of the immobilized antibody was inevitable.

**Figure 3.**
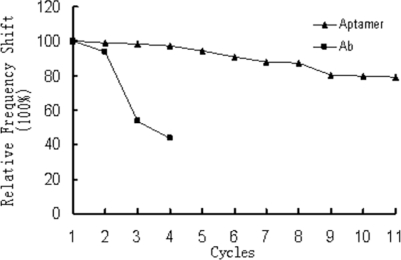
Comparison of the regeneration between aptamer-based biosensor and antibody-based biosensor by EDTA. IgE (100 μg/L) was applied to the gold surface for detection. The regeneration reagent was 30 mmol/L EDTA solution. The relative frequency shift (%) was the frequency shift measured relative to the response for the first measurement. The cycles of x-axis mean the number of the regeneration test.

Unlike the antibody-based biosensor, the aptamer-based biosensor could be denatured and reused many times on the same chip, without loss of function. Binding of IgE to the aptamers was an almost completely reversible procedure. As shown in Figure 3, using 30 mmol/L EDTA as regeneration reagent led to dissociation of the analyte. The aptamer layer being completely reconstituted simply by returning to original buffer conditions (PBS). The crystals could be used continuously for 10 times, and the relative frequency shifts obtained were all more than 80% of the response obtained at the first cycle. The regeneration procedure also had been proved to be simplistic with just EDTA. This is because IgE aptamers need bivalent metal ions to assemble into their three-dimensional structure and unfold in the presence of EDTA. They refold very rapidly when EDTA is withdrawn and replaced by Mg^2+^-containing buffer [20].

Aptamer or antibody-coated crystals were covered with 0.02% sodium azide solution in PBS and stored at 4 °C for long-time stability test. We made three different crystals for measurement per day continuously for 35 days. At the end of the third week, the loss of adsorbed surface mass was calculated to be only 10%, indicating loss of probes was negligible over this time period for aptamer-coated crystals. For antibody-coated crystals, the stability was almost fully maintained at the first two days and then started to decline. After one week, the relative frequency shifts of antibody-coated crystals were about 40% compared with its initial response. This is because that the antibodies are sensitive to temperature and undergo irreversible denaturation. The antibodies also have a limited shelf life. The reusability and long-time stability of the aptamer-based biosensor showed that the cost of detection could be reduced by this method.

## 3. Experimental Section

### 3.1. Reagents

Avidin, 3,3′-dithiodipropionic acid di(*N*-succinimidyl ester) (DSP), *N,N*-dimethylacetamide (DMA), staphylococcal protein A (SPA), and lysozyme were purchased from Sigma (St. Louis, MO, USA). Bovine serum albumins (BSA) and human serum albumins (HSA) were purchased from Roche (Indianapolis, IN, USA). IgE, IgG and IgM were purchased from US Biological Company (Boston, Massachusetts, USA). All the other reagents were purchased from Sigma (St. Louis, MO, USA). Monoclonal anti-human IgE antibody was purchased from Beckman (St. Louis, MO, USA). The composition of the buffers used for the experiments is reported below:

Immobilization buffer: 10 μM Tris-HCl buffer, 0.2 M NaCl, pH 7.9.
Reaction buffer: 8.1 mM Na_2_HPO_4_, 1.1 mM KH_2_PO_4_, 1 mM MgCl_2_, 2.7 mM KCl, and 138 mM NaCl, the pH was adjusted to 7.4, which was based on the reported SELEX conditions [29].

The base sequence for the anti-IgE aptamer (D17.4) has been found to be 5′-GGGGCACGTTTAT-CCGTCCCTCCTAGTGGCGTGCCCC-3′, as noted from the initial work of Wiegand and co-workers [31]. The aptamer with the 5′-modified with biotin was custom designed and purchased from Shanghai Bioengineering Company (Shanghai, China).

### 3.2. Apparatus

The 10 MHz AT cut quartz crystal (13 mm × 13 mm) with gold evaporated on both sides was obtained from 26th Research Institute, Chinese Electronic Scientific and Technical Group Company (Chongqing, China). The 2 × 5 model micro-array sensor was made by China Jialing Group (Chongqing, China). The frequency variations were continuously recorded using a frequency data acquisition card (Model PCL-836, Yanhua Co., Taiwan, China): the data (the resonance frequency) was displayed on the display screen and could be read directly by a computer connected to the PCL-836 interface. The frequency shifts reported were the difference between two stable frequency values (±1 Hz). The cell was used in static conditions. Temperature was controlled (≤1 °C) by an air thermostat. The measuring apparatus consisted of the crystals fixed inside the sensor, only one side of which was exposed to the solutions. All detection wells were designed with the same structure, and each of them was driven by an independent logic circuit so that they worked independently, without mutual interference. The electrode surface was soaked and rinsed for 10 min in cleaning solution (30% H_2_O_2_: 98% H_2_SO_4_ = 1:3) and then thoroughly washed with tri-distilled water and dried with pure nitrogen gas.

### 3.3. Immobilization of Aptamer

Immobilization of aptamers on gold-coated quartz crystals was performed as follows: the prepared gold surfaces were activated by injection with 10 μL of 4 mg/mL DSP in water-free DMA, and incubated at room temperature for 15 min. The gold surfaces were then washed three times with PBS, followed by the immediate injection with 10 μL of 2 mg/mL avidin in PBS, and incubated at 4 °C overnight. The 5′-biotin-labeled aptamer solution at a concentration of 2 μmol/mL was heated to 95 °C for 3 min and chilled on ice to place the aptamer in its favorable thermodynamic conformation. Subsequently, 10 μL of aptamer solution was added onto the gold surface of the crystals, and incubated at room temperature for 1 h. Unbounded aptamers were removed by rinsing with PBS buffer three times. To block unreacted surface groups, 10 μL of 0.025% BSA solution was applied; the slides were then incubated at room temperature for 1 h and rinsed by PBS buffer three times. Aptamer-coated crystals were either used immediately or covered with 0.1% sodium azide solution in PBS and stored at 4 °C.

### 3.4. Immobilization of Antibody

First, 5 mL of 1 mg/mL SPA solution in PBS was injected on the gold surface of the crystals for 20 min, following by a through rinse with PBS buffer to remove the unreacted SPA. Then 10 μL of 5 mg/mL anti-IgE antibody was added on the gold surface of the crystals after the activation with SPA. After 20 min the antibody was washed off and the remaining activated sites of the layer were saturated with BSA as described in Section 3.3.

### 3.5. Detection Procedures

Ten prepared crystals were placed into the detection wells with the side coated with aptamer (or antibody) facing up to construct the 2 × 5 model array. The system temperature was adjusted to 37 °C. Then, 90 μL of reaction buffer was added to each cell of the sensor. The resonance frequency (F_0_) was monitored until a steady baseline was obtained. Then 10 μL IgE standards of different concentrations (2.5–250 μg/L) were added into the wells respectively. PBS buffer was added to chamber 10 as a negative control. When the resonance frequency reach a stable value, that means the reaction between the aptamer (or antibody) and the IgE came to an end. Then, another steady state resonant frequency was taken as F_1_. The frequency shifts (ΔF = F_1_ – F_0_) induced by aptamer-IgE (or antibody-IgE) were the difference between the final value (F_1_) and the value displayed before reaction (F_0_). The equilibrium time for reaction was also recorded. The average value was taken by repeating the experiment 3 times. The dose-curve was drawn according to the relationship between frequency shifts and IgE concentrations. Human IgG, IgM, and HSA (Sigma) were used as nonspecific binding controls. All proteins were dissolved in a PBS buffer at a concentration of 500 mg/mL, which served as a stock solution.

### 3.6. Regeneration Procedures

The regeneration of the crystals used is an important factor to be considered in the development of a practical biosensor. The regeneration solution, 0.2 mmol/L glycine/HCl (pH 2.8), 1.2 mmol/L NaOH, and 30 mmol/L EDTA, were applied on the electrodes for two minutes to denature the aptamer or antibody and remove the IgE from it after each measurement. The obtained surfaces were then rinsed with PBS buffer three times. After washing with tri-distilled water the frequency reached a stable value (±1 Hz) with buffer, aptamer or antibody once again responded to the IgE and a new detection step could be performed.

### 3.7. Materials Methodology Comparison

First, we generated the calibration curves by plotting ΔF against various IgE concentrations, and the linearity was determined by serially diluting the stock IgE solutions. After the analysis procedures, the operational characteristics, including imprecision, accuracy, specificity, analytical sensitivity, and detection limit, were evaluated. Then, the serum samples were quantified individually with the QCM biosensor and chemiluminescence (Access Immunoassay System, Beckman). All participants gave signed informed consent and the study was approved by the Ethics Boards of the Third Military Medical University. Bland-Altman analyses were performed to evaluate the residuals plots of the proposed methods and reference method.

### 3.8. Statistical Treatment

The results are expressed as mean (±SD). SPSS 10.0 software was used for the *t*-test, and the Bland-Altman analyses were performed with GraphPad, version 5.0.

## 4. Conclusions

Antibodies and aptamers can bind to their targets with high affinity and specificity, aptamers offer distinct advantages over antibodies that make them very promising in analytical and diagnostic applications. These advantages include the easily regenerating the function of the immobilized aptamers and the possibility of using different detection methods due to easy labeling [32,33]. Due to their high binding affinity, simple synthesis, easy storage, and wide applicability, aptamers are emerging as a new class of molecules that rival commonly used antibodies in protein recognition and detection.

Here we assessed a DNA-aptamer as an affinity ligand, and it compared favorably with its antibody counterpart. Compared to antibodies (10 ng/mL), aptamer-based analyte recognition was at least as sensitive. Aptamers could detect their analytes (IgE) at a minimum concentration of 2.5 ng/mL. Regarding specificity, aptamers were even superior to antibodies, which exhibited higher signal backgrounds due to a higher degree of nonspecific protein binding. Moreover, for clinical sample detection, the consistency between QCM aptamer biosensor and chemiluminescence method is good. Another advantage over antibodies can be seen in the higher stability of aptamers, as aptamers are very stable and they can recover their native active conformation after denaturation. The results suggested that the aptamer-based biosensor was sensitive, specific, and easy-operating method. These results raise the prospect that aptamers could be adapted to function as reusable, long-time biosensors in arrays.

Recently, there has been an interest in biosensors with aptamers as the bio-recognition element. In most cases, only limited quantities of IgE from standards are available by QCM biosensor. To achieve the object of detecting IgE in clinical serum samples, we combined QCM and aptamer together. QCM has high sensitivity and aptamer has high selectivity. Combining the two elements together, sensitivity was greatly enhanced, and detection can be performed directly on clinical serum samples. Our results showed that this system can detect about 2.5 ng/mL IgE, making it a feasible assay for practical applications. In conclusion, this aptamer-based QCM biosensor can directly detect IgE from clinical serum samples with high sensitivity and selectivity. It may be a rapid, efficient and simple approach for detection of IgE in clinical specimens. Given that QCM immunosensor analyses are fairly rapid and relatively easy to operate, this approach lends itself particularly well to the rapid development of an analytical method in clinical laboratories.

## Figures and Tables

**Table 1.** Summary of the IgE determination limit obtained by various methods.

**Method [reference]**	**Detection limit**
QCM	50 pM
CE [26]	46 pM
ELISA [27] ^a^	45 pM
Molecular Light Switch Complex [28]	100 pM
Fluorescence Anisotropy [29]	350 pM
Electrochemical Sensor [30]	158 pM

a ELISA method used antibody as bio-recognition element; other methods used aptamer as bio-recognition element.
